# Whole-Genome Sequence of Listeria newyorkensis, Isolated from River Water in Japan

**DOI:** 10.1128/MRA.00488-19

**Published:** 2019-08-15

**Authors:** Chihiro Ohshima, Hajime Takahashi, Ayaka Nakamura, Takashi Kuda, Bon Kimura

**Affiliations:** a Department of Food Science and Technology, Faculty of Marine Science, Tokyo University of Marine Science and Technology, Tokyo, Japan; Indiana University, Bloomington

## Abstract

Listeria newyorkensis is a bacterium that was recently classified into the genus Listeria. So far, there are only two reports of isolation of L. newyorkensis from food. Here, we report the whole-genome sequence of *L. newyorkensis* strain 2-1, isolated from river water in Japan.

## ANNOUNCEMENT

Listeria newyorkensis is a Gram-positive, facultative anaerobic, rod-shaped bacterium (1). Two of the isolated L. newyorkensis strains have been cultured from a seafood processing plant and raw milk (1, 2). As there are a limited number of reports related to this bacterium, its distribution has yet to be comprehensively elucidated.

In a surveillance study conducted by our lab in Japan between 2007 and 2013, we isolated a strain of *Listeria* from river water, and it was identified as *L. newyorkensis*. The isolate that was finally identified as *L. newyorkensis* during this survey was obtained. Here, we report the whole-genome sequence of this strain.

The surveillance study was conducted at the Kikuchi River, which is located in Kyushu in the western region of Japan. We collected a liter of river water and tested it for the presence of *Listeria* spp. We isolated the bacteria from the water using a 0.22-μm cellulose acetate membrane. The membrane was transferred into 100 ml of Half Fraser broth and incubated for 24 h at 30°C. After incubation, 1 ml of the Half Fraser broth was transferred to 9 ml of Fraser broth and incubated at 30°C for 24 h. Next, the cultured broth was streaked onto a polymyxin acriflavin lithium chloride ceftazidime esculin mannitol (PALCAM) agar plate. The plate was incubated at 30°C for 48 h. Finally, the colonies of *Listeria* spp. were isolated. These colonies were identified by comparing the 16S rRNA sequences of Listeria rocourtiae CIP109804^T^, *L. newyorkensis* FSL M6-0635^T^, and Listeria cornellensis DSM26689^T^. One of the colonies was identified as *L. newyorkensis* based on the identity of its 16S rRNA sequences with that of *L. newyorkensis* (1).

　Genomic DNA was extracted from the bacteria cultured in brain heart infusion (BHI) broth using a previously described method (3), and the isolated genomic DNA was treated as the library for whole-genome sequencing, which was done using the Ion Xpress Plus fragment library kit. Next, the sample was loaded onto the Ion 318 Chip with the Ion Chef system and sequenced using the Ion personal genome machine (PGM) sequencer.

After sequencing, low-quality reads were filtered out, and the barcode sequences were trimmed using the Torrent server software. In total, 6,095,054 reads were generated. The average read length was 294 bp. Then, the SPAdes assembler version 5.8.0.0 software was used to create the contigs. The assembly was assessed based on the number of contigs, GC content, *N*_50_, and total length using the QUAST v.2.3 software (4). Finally, 55 contigs (≥500 bp) were generated. The lengths of these contigs ranged from 560 to 345,450 bp, and the total length of all the contigs was 3,421,445 bp (GC content, 43.03%). The *N*_50_ value was calculated as 124,333 bp. Default parameters were used for all software, unless otherwise specified.

*Listeria* spp. have often been isolated from food and other aspects of the environment, such as soil (5–7). To date, *L. newyorkensis* strains have been isolated only from food sources (1, 2). However, our surveillance study showed that this strain can also be found in other environments.

There is limited information regarding the detection of newly registered *Listeria* spp., in particular, those belonging to the *Listeria sensu lato* group, resulting in a lack of information about the genome sequence and the distribution area of this species. Therefore, the information related to not only L. monocytogenes but also other *Listeria* spp. is required for defining the index for species identification and elucidating the phylogeny of the *Listeria* genus.

### Data availability.

The assembled genome sequence was registered in GenBank under the accession number BJEY00000000. The version described in this paper is BJEY01000000. Raw sequence reads have been deposited in the NCBI Sequence Read Archive under the BioProject number PRJDB7923 and under accession number DRR165934.
